# Modulation of out-of-plane reflected waves by using acoustic metasurfaces with tapered corrugated holes

**DOI:** 10.1038/s41598-019-52441-w

**Published:** 2019-11-01

**Authors:** Xiao-Shuang Li, Yan-Feng Wang, A-Li Chen, Yue-Sheng Wang

**Affiliations:** 1 Institute of Engineering Mechanics, Beijing Jiaotong University, Beijing, 100044 China; 2 School of Mechanical Engineering, Tianjin University, Tianjin, 300072 China

**Keywords:** Acoustics, Structure of solids and liquids

## Abstract

In this paper, modulation of reflected wavefront out of the incident plane by a tunable acoustic metasurface is investigated based on the fully generalized Snell’s law in the three-dimensional space. The metasurface is constructed by a square lattice of circular holes with gradient annular bumps. The phase shift is tuned by changing the volume of water filled in the holes. The acoustic wave steering out of the incident plane and the out-of-plane acoustic focusing with the oblique incidence at the subwavelength scale are demonstrated numerically by selecting suitable distributions of water depth. The numerical results show that the wavefront of the reflected wave can be manipulated over a wide frequency range; and the gradient design of the unit cells can suppress the parasitic reflection. The present work is relevant to the practical design of novel acoustic devices.

## Introduction

Recently, the metasurface, as a thin layer with gradient distribution of subwavelength micro-structures, has received considerable attentions^1^. The existence of a discontinuous phase makes it possible to modulate the wavefront of the reflected or refracted waves based on the generalized Snell’s law (GSL)^2,3^. The concept of metasurfaces was first proposed in the field of electromagnetic waves. According to the GSL, the anomalous propagation of electromagnetic waves across a material interface can be predicted^4^. By redistributing the discontinuous phase, electromagnetic waves can be engineered to achieve extraordinary functionalities such as bending a beam along an arbitrary trajectory^5^, converting an incident bulk wave into a surface mode^6^, manipulating the polarization^7^, and constructing ultrathin flat lens that focus light beams^8–10^, etc.

As a kind of classical waves, acoustic waves also obey the GSL^11^. As the analogue of optical metasurfaces, acoustic metasurfaces were recently proposed to manipulate the wavefront of the transmitted or reflected wave. In order to redistribute the phase, a straightforward method is to change the propagating distance of the wave in the micro-structures of the metasurfaces. The most successful design is the coiling space structures, such as the labyrinthine structure^11^ or the system composed of two stiff corrugated arms^12^. In these metasurfaces, the incident acoustic wave is restricted to propagate in the coiling channel, resulting in a phase delay. By introducing different coiling space units, an arbitrary phase distribution can be implemented. Fascinating properties of acoustic waves have been demonstrated both numerically and experimentally^13^, e.g. anomalous reflection and flat lenses^14–18^, self-accelerating beams^19^, negative and zero effective refractive indices^20^, wave mode conversion^21^, cloaking^22,23^, and super absorption^24^, etc. Recently, several measures have been taken to improve the performance of metasurfaces. For instance, almost perfect reflection/refraction was achieved by using a power-conformal metamirror^25^ or considering the non-local response^26^; and the limitations of the local designs were broken by adapting a nonlocal passive metasurface^27^.

It is noted that most investigations focus on two-dimensional modulation of reflected and/or refracted waves in the incident plane, or on axial modulation such as focusing and imagine^28^, Airy-beam^29^ and bottle beam^19^, etc. Research on the modulation of the reflected acoustic waves out of the incident plane is rarely reported. Zhao *et al*.^30^ studied the modulation of the out-of-plane reflected acoustic waves based on the so-called impedance-governed GSL. The reflected waves were tailored by adjusting the distribution of the specific surface impedance. In contrast, adjusting the phase distribution of a complex structure is easier in practice. In fact, when the phase distribution of a metasurface is gradient along an arbitrary direction, the incident wave will be reflected out of the incident plane. As far as we know, Aieta *et al*.^31,32^ first considered the out-of-plane reflection and refraction of electromagnetic waves and derived the mathematical expression of the fully three-dimensional GSL (3D-GSL) by means of Fermat’s principle.

In this paper, we propose a tunable tapered acoustic metasurface to control the out-of-plane reflection of acoustic waves. The elementary unit is a corrugated cylindrical hole with tapered annular bumps. The gradient design of the bumps is used to suppress parasitic reflections (i.e. the reflected waves in unwanted directions) originating from the strong scattering near the metasurfaces. Tunability is realized by filling liquid (water) into the holes. The phase of reflected waves in air can be tuned by changing the volume of the liquid. A phase shift of a 2*π* span is obtained. This provides a possibility to freely manipulate the wavefront of the reflected waves. As illustrations, anomalous out-of-plane reflection of acoustic waves and three-dimensional out-of-plane acoustic focusing with the oblique incident waves are implemented.

## Design of the Fundamental Unit Cell

**Figure 1:**
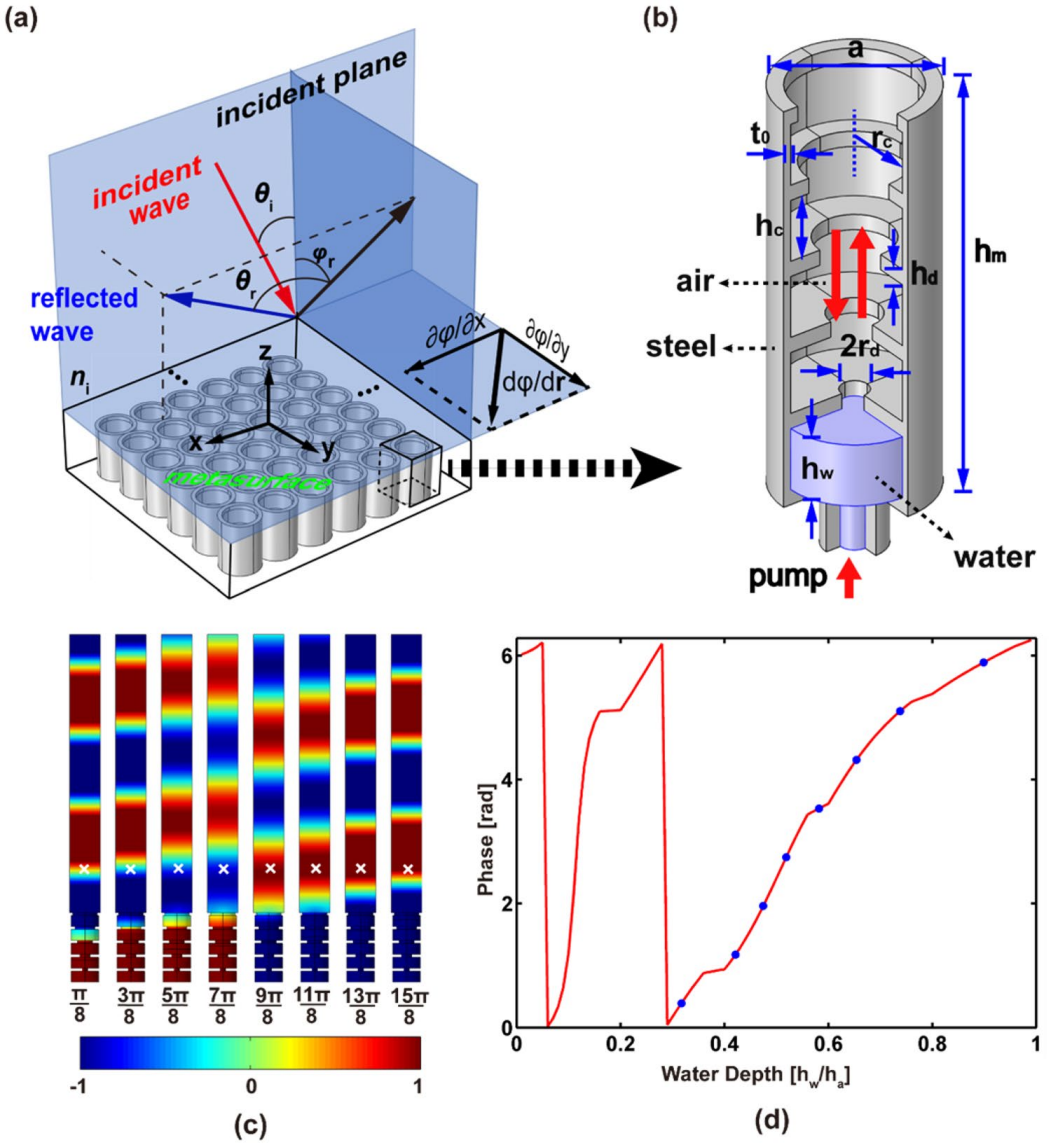
The fundamental unit and the 3D-GSL. (**a**) The 3D-GSL and the metasurfaces. (**b**) The fundamental unit filled by water. The water is injected from the bottom of the unit cell through a pump. (**c**) The normalized pressure fields of reflected waves are shown for some selected water depths. The corresponding phases are marked by blue dots in panel (d). The marker “×” indicates the location for retrieve the phase shift. The color scale indicates the amplitude of the pressure field from negative (blue) to positive (red). (**d**) The reflection phase shift is plotted as a function of water depth at wavelength *λ* = 5*a*.

The proposed metasurface is composed of a steel plate, in which corrugated cylindrical holes with tapered annular bumps are distributed in a square array, as shown in Fig. 1(a). The elementary unit is depicted in Fig. 1(b). The diameter of the unit is *a*; the thickness of the wall is *t*_0_; and the height is *h*_*m*_. The depth of the hole is *h*_*a*_ = *h*_*m*_ − *t*_0_. The annular bumps with thickness *h*_*d*_ divide the hole into several cavities with height *h*_*c*_. The water is supposed to be injected from the bottom of the unit cell through a pump. The phase delay of the incident acoustic wave propagating into and back out of the hole can be tuned by changing the water depth *h*_*w*_. By filling the holes with different volumes of water, a gradient phase distribution in the metasurface can be obtained. In this way, a tunable metasurface can be designed^33–36^ and programmable tunability can be realized^37^.

As demonstrated in refs^38,39^ the tapered unit cells, which are gradient in the thickness direction of the metasurface, can improve the impedance matching^38^ and suppress the parasitic waves^39^. Following this idea, here in the present paper, we propose a gradient design of the unit cell with the inner radius of the bumps *r*_*d*_ decreasing linearly from top to bottom to suppress parasitic scattering. The fundamental unit cell with a horn-shaped inner structure is shown in Fig. 1(b). We have checked that if all bumps are of the same size, the parasitic waves propagating to unwanted direction is so strong that we can hardly get a purely out-of-plane reflected wave, especially for a small incident angle (see Supplementary Material for details).

The phase shift varying with the water depth is the most important relation in metasurface design. Here the finite element (FE) method is applied to obtain this relation numerically. Simulations are implemented by using the software COMSOL Multiphysics. The solid structure (steel) is considered as a rigid body. The FE model is composed of a fundamental unit cell and an additional block acting as the background field. The Plane Wave Radiation (PWR) boundary condition is applied on the top of the background field. The periodic boundary condition is applied on the lateral sides of the block. The specific geometric parameters of the fundamental unit cell in the following computation are assumed to be *a* = 10 mm, *h*_*m*_ = 27 mm, *t*_0_ = 1 mm, *h*_*d*_ = 1 mm and *h*_*c*_ = 4 mm. The values of the inner radius *r*_*d*_ that follow a gradient variation are selected as 3.996 mm, 3.222 mm, 2.448 mm, 1.674 mm, and 0.9 mm. Both air and water are considered as the acoustic media supporting linear pressure waves. The velocities of the air and water are 343 m/s and 1490 m/s. The densities of the air and water are 1.21 kg/m^3^ and 1000 kg/m^3^ respectively. The wavelength of the incident wave is assumed to be *λ* = 5*a*. The coupling between the unit cells is neglected^40^. The pressure fields for some selected values of the water depths are shown in Fig. 1(c). The phase information is retrieved at a point 2*a* away from the top of the unit cell in order to avoid the deviation originating from inhomogeneous field near the metasurface. Figure 1(d) shows the relation of the phase shift varying with the water depth from 0 to 2*π*. This satisfies the requirement for a full control of acoustic waves^1^.

In the following, we will demonstrate the manipulation of the different out-of-plane reflected wavefront by redistributing the water depths at the subwavelength scale. Without loss of generality, we assume that the metasurface is in *XY*-plane, and the incident plane is *XZ*-plane, see Fig. 1(a). The incident wavelength is *λ* = 5*a* without further specification.

### Modulation of out-of-plane reflection

When an acoustic wave is obliquely incident on the metasurface with the gradient phase distribution along an arbitrary direction, the out-of-plane reflection may appear. This anomalous phenomenon can be described by the 3D-GSL which is illustrated in Fig. 1(a). Suppose the *XY*-plane is on the upper surface of the metasurface, and the incident plane is *XZ*-plane with the incident angle being *θ*_*i*_. The reflected beam makes an angle of *θ*_*r*_ with respect to *YZ*-plane, and its projection on *YZ*-plane makes an angle of *φ*_*r*_ with respect to *XZ*-plane. According to the Fermat’s principle, the distribution of the phase shift should satisfy the following 3D-GSL^31,32^:

1 $$\sin \,{\theta }_{{\rm{r}}}-\,\sin \,{\theta }_{{\rm{i}}}={({n}_{0}{k}_{0})}^{-1}\partial \Phi /\partial x,$$

2 $$\cos \,{\theta }_{{\rm{r}}}\,\sin \,{\phi }_{{\rm{r}}}={({n}_{0}{k}_{0})}^{-1}\partial \Phi /\partial y,$$

where the phase profile Φ is to be determined as a function of (*x*,*y*); *n*_0_ is the refraction-index in the air; and *k*_0_ is the wave number. By combining Eqs (1) and (2), the phase profile for the metasurface can be obtained as

3 $$\Phi (x,y)={n}_{0}{k}_{0}[(\sin \,{\theta }_{{\rm{r}}}-\,\sin \,{\theta }_{{\rm{i}}})x+\,\cos \,{\theta }_{{\rm{r}}}\,\sin \,{\phi }_{{\rm{r}}}y]$$

The above equation implies that the reflected waves can be tailored freely by engineering the phase distribution carefully along the *x*- and *y*-directions. More specifically, for arbitrarily given reflection angles *θ*_*r*_ and *φ*_*r*_, the distribution of the phase shift can be determined from Eq. (3). Then the corresponding distribution of the water depth can be obtained from Fig. 1(c). Finally, the metasurface with the target function is configured.

To demonstrate the feasibility of the proposed design, we construct a metasurface using a square lattice of 16 × 16 unit cells. In the FE model, a plane wave with a unit amplitude is assumed as the area source. The Perfect Matched Layer (PML) is applied to eliminate the boundary reflection. In order to ensure the accuracy of the calculation, the maximum mesh size is set as *a*/8.

**Figure 2:**
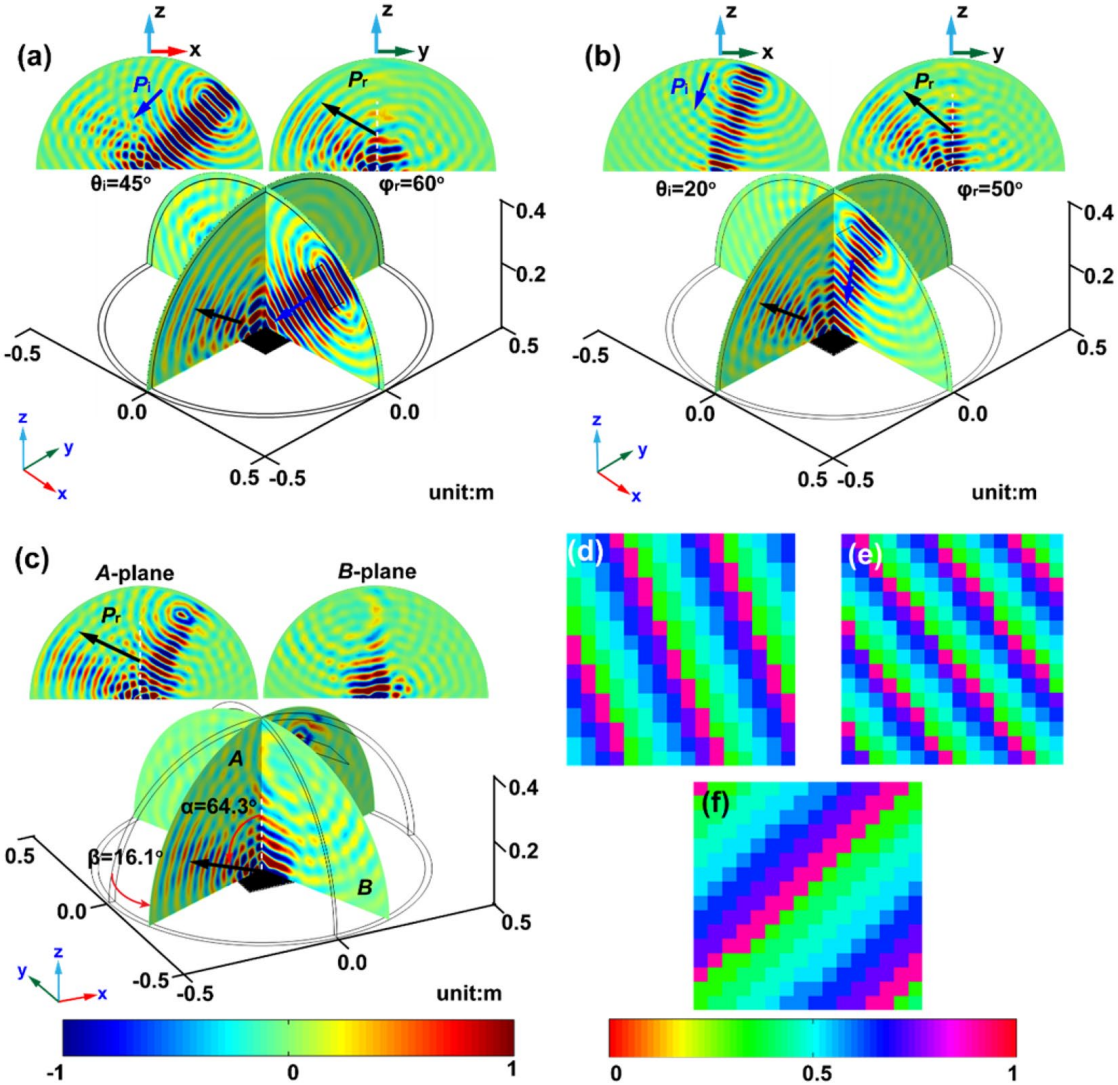
Tunable out-of-plane reflection. The reflected pressure fields are shown for anomalous reflections with (**a**) *θ*_*i*_ = 45°, *θ*_*r*_ = 0°, *φ*_*r*_ = 60°, (**b**) *θ*_*i*_ = 20°, *θ*_*r*_ = 0°, *φ*_*r*_ = 50° and (**c**) *θ*_*i*_ = 30°, *θ*_*r*_ = 60°, *φ*_*r*_ = 45° or equivalently, *α* = 64.34° and *β* = 16.10°. The inset figures illustrate the reflection plane (*YZ*-plane or *A*-plane) and the incident plane or the plane perpendicular to the reflection plane (*B*-plane). The blue arrows indicate incident waves; and the black ones indicate reflected waves. The color maps indicate the acoustic pressure. Since *A*-plane is not perpendicular to the incident plane, so the pressure field of the incident wave is observed in *A*-plane. The corresponding distributions of water depth are illustrated in (**d**–**f**) with the color map indicating the water depth.

Figure 2 presents three examples of anomalous reflection by the tunable metasurface. We first consider the special case that the reflection plane is perpendicular to the incident plane. This means the reflected beam is in *YZ*-plane, i.e. *θ*_*r*_ = *θ*°. Then Eq. (3) can be simplified as

4 $$\Phi (x,y)={n}_{0}{k}_{0}[-\sin \,{\theta }_{{\rm{i}}}x+\,\sin \,{\phi }_{{\rm{r}}}y].$$

In the first example, we consider the incident wave with the angle *θ*_*i*_ = 45° imping on the metasurface. The targeted reflect direction is *φ*_*r*_ = 60° in *YZ*-plane. Then the water depth in every fundamental unit cell of the metasurface is determined by Eq. (4) and Fig. 1(c). The distribution of the water depth is shown in Fig. 2(d). The scattered pressure field calculated by FE method is shown in Fig. 2(a). It is observed that the reflected waves in the incident plane (*XZ*-plane) decay rapidly away from the metasurface within a distance about three times of wavelength. The black arrow in *YZ*-plane in Fig. 2(a) indicates the propagation direction of the reflected waves. It forms an angle of 60° with respect to the *z*-axis, which is consistent with the purpose of the design. In the second example, the incident wave with *θ*_*i*_ = 20° in *XZ*-plane is expected to be converted to the reflected wave with *φ*_*r*_ = 50° in *YZ*-plane. The distribution of the water depth in the metasurface is obtained by following the same procedure, and shown in Fig. 2(e). The simulated scattered pressure field in Fig. 2(b) is in line with our design. It is worth notice that the parasitic reflections in the incident plane almost disappear, even when the incident angle is small. This advantage is attributed to the gradient design of the fundamental unit cells.

As we mentioned before, the proposed metasurface can be used to freely redirect the reflected waves. As the third example, we consider a general case that the reflected beam is modulated to propagate in an arbitrary direction. Two auxiliary angles, *α* and *β*, are defined to describe the orientation of the reflected wave, where $\alpha ={\cos }^{-1}(\cos \,{\theta }_{{\rm{r}}}\,\cos \,{\phi }_{{\rm{r}}})$ is the angle of the reflected wave with respect to *z*-axis, and $\beta ={\sin }^{-1}(\sin \,{\phi }_{{\rm{r}}}\,\cos \,{\theta }_{{\rm{r}}}/\sqrt{{\sin }^{2}{\phi }_{{\rm{r}}}{\cos }^{2}{\theta }_{{\rm{r}}}+{\sin }^{2}{\theta }_{{\rm{r}}}})$ is the angle between the reflection direction and the incident plane. For illustration, we set the incident angle as *θ*_*i*_ = 30° and reflected angles as *θ*_*r*_ = 60° and *φ*_*r*_ = 30°. Thus, we have *α* = 64.34° and *β* = 16.10°. The distribution of the water depth, determined by Eq. (3) and Fig. 1(c), is shown in Fig. 2(g). The simulated scattered pressure field is plotted in Fig. 2(c). The propagation direction of the reflected wave is found to be in excellent agreement with the target.

It is further noted that the distribution of the water depth in Fig. 2(d–f) are linear. To explain this, we rewrite Eq. (3) as

5 $$\Phi (x,y)={n}_{0}{k}_{0}(y+\kappa x),$$

with $\kappa =\frac{\sin \,{\theta }_{{\rm{r}}}-\,\sin \,{\theta }_{{\rm{i}}}}{\cos \,{\theta }_{{\rm{r}}}\,\sin \,{\phi }_{{\rm{r}}}}$. Equation (5) indicates that the unit cells with the same phase (or the same water depth) should be located in a straight line with the slope of −*κ* which is determined by the incident and reflected angles. This issue is confirmed by the distribution of the water depth shown in Fig. 2(d–f).

In summary, the designed tunable metasurface can modulate the reflected wave propagation out of the incident plane. And it is also shown that the parasitic reflection successfully suppressed. Here we indicate that the suppression of the parasitic waves is owning to the gradient design of the fundamental unit cell other than the sample size. To demonstrate this, we compute larger samples with 24 × 24 unit cells. The results show that the parasitic waves still exist for a large metasurface if the fundamental unit cells have no tapered structures. Detailed analysis can be found in the Supplementary Material.

### Arbitrary 3D focusing of reflected wave

**Figure 3:**
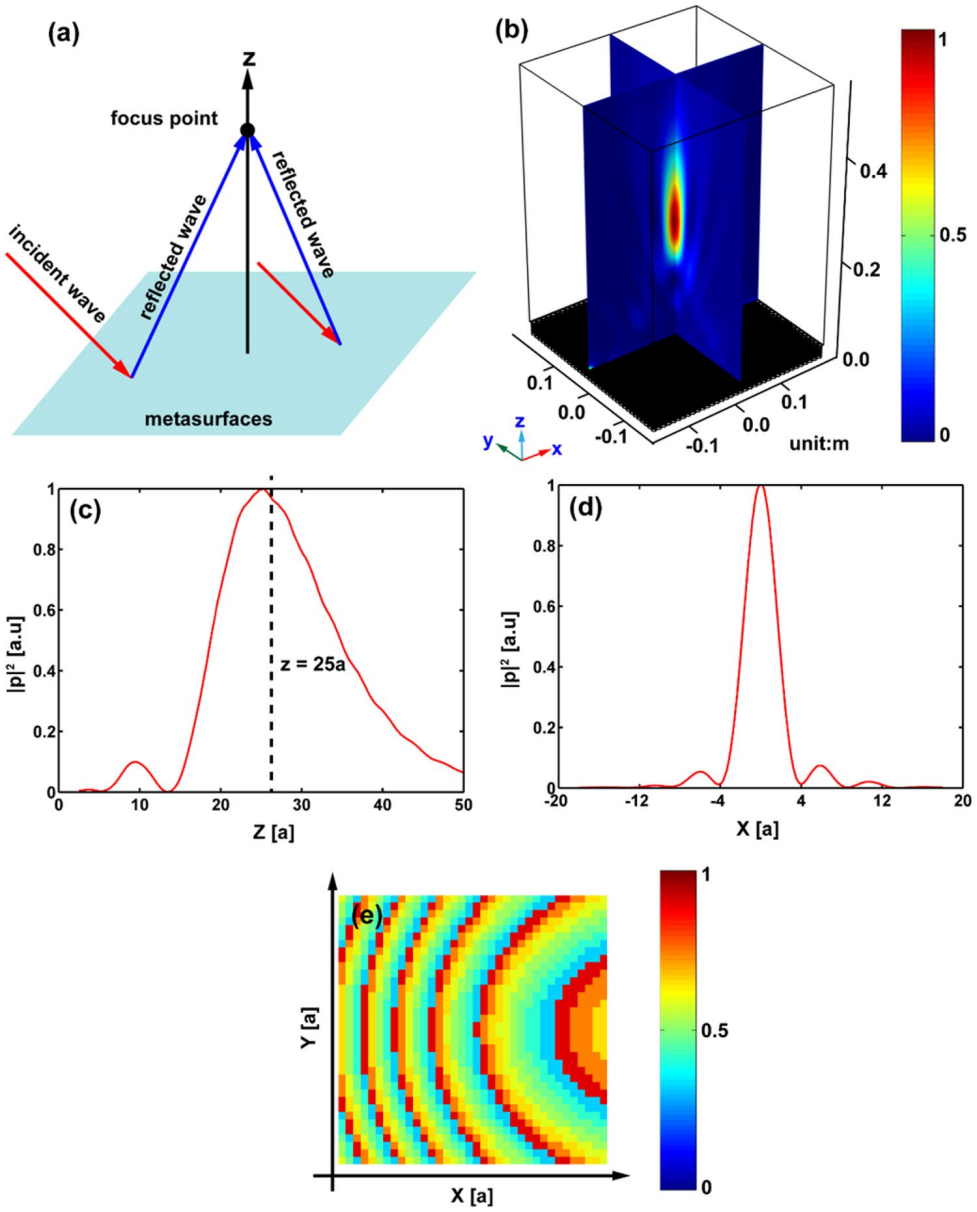
3D axial focusing for reflected waves. Panel (a) shows the reflected waves focusing on an arbitrary point for arbitrarily incident waves. Panel (b) illustrates the normalized intensity distribution ${|p|}^{2}$ of the reflected waves, which is represented by the color scale. (**c**) Shows the cross sections of the normalized intensity profile along *z*-axis. The peak appears at *z* = 25*a*(*z* = 25 cm). (**d**) Shows the transverse cross section of the intensity field at *z* = 25*a*. (**e**) Shows the distribution of water depth in the metasurface.

Metasurfaces provide many probabilities to design a variety of acoustic devices with different applications. Here we will construct a flat lens for reflected waves using the designed tunable metasurface. The 3D-GSL should be applied in the general case where the reflected waves of arbitrarily incident waves are focused on an arbitrary point in the three-dimension space, see Fig. 3(a).

We first consider a simple case that the focus point is located on the *z*-axis, the phase profile can be deduced from Eqs (1) and (2) as

6 $$\Phi (x,y)={n}_{0}{k}_{0}(\sqrt{{x}^{2}+{y}^{2}+{z}_{{\rm{f}}}^{2}}-{z}_{{\rm{f}}}-x\,\sin \,{\theta }_{{\rm{i}}}).$$

It should be emphasized that Eq. (6) is only an approximation limited to the second order^9^, but it is enough for most applications. For verification, a flat lens with 36 × 36 unit cells arranged in a square lattice is designed. The focus point is supposed at *z*_*f*_ = 25*a* and the incident angle is *θ*_*i*_ = 45°. The corresponding distribution of the water depth is illustrated in Fig. 3(e). Figure 3(b) presents the field distribution of the spatial intensity ${|p|}^{2}$. To measure the location of the focus point, the cross sections of the intensity of the reflected acoustic field along the *z*- and *x*-axes are shown in Fig. 3(c,d), respectively. The focus point is observed to be located at *z* = 25*a* (25 cm), in excellent agreement with the target. The full width at a half-maximum (FWHM) in Fig. 3(d) is about 0.72 *λ*, implying subwavelength focusing. The intensity at the focus spot in Fig. 3(d) is nearly 13.25 times larger than that of the incident waves.

Next we consider a general case for an arbitrary focal point at (*x*_*f*_, *y*_*f*_, *z*_*f*_). In this case, the corresponding phase profile can be obtained as:

7 $$\Phi (x,y)={n}_{0}{k}_{0}(\sqrt{{(x-{x}_{{\rm{f}}})}^{2}+{(y-{y}_{{\rm{f}}})}^{2}+{z}_{{\rm{f}}}^{2}}-{z}_{{\rm{f}}}-x\,\sin \,{\theta }_{{\rm{i}}}).$$

**Figure 4:**
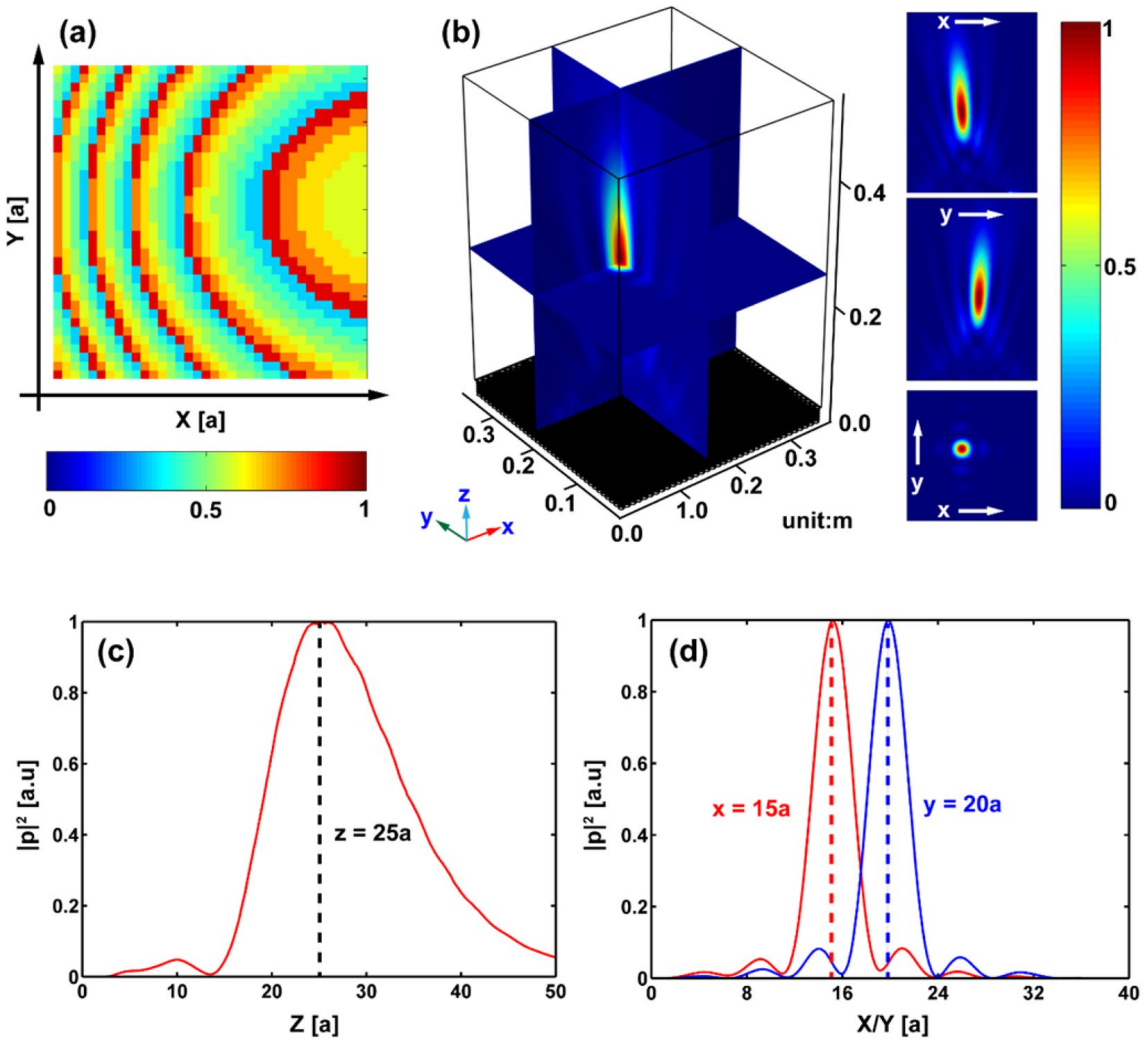
3D arbitrary focusing for reflected waves. Panel (a) shows the distribution of the water depth in the metasurface. Panel (b) shows the intensity distribution ${|p|}^{2}$ of the reflected waves field. The inset panel illustrates the intensity distribution ${|p|}^{2}$ on *XZ*-, *YZ*- and *XY*-planes. The normalized intensity ${|p|}^{2}$ is represented by the color scale. (**c**) Shows the cross section of the normalized intensity profile along *z-*direction with *x* = 15*a* and *y* = 20*a*. The peak appears at *z* = 25*a*(=25 cm), which is in very good agreement with the target value we designed. (**d**) Shows the transverse cross section of the normalized intensity profile along *x*-direction (red line) and *y*-direction (blue line) at *z* = 25*a*.

Obviously, when x_f_ = 0 and y_f_ = 0, Eq. (7) is reduced to Eq. (6). In order to demonstrate Eq. (7), we tune the distribution of the water depth [see Fig. 4(a)] of the above designed metasurface to focus the incident beam with *θ*_*i*_ = 45° at the focus point (15*a*, 20*a*, 25*a*). The intensity field ${|p|}^{2}$ is shown in Fig. 4(b). For clarity, the cross sections of the intensity fields on *XY*-, *XZ*- and *YZ*-planes are also presented. The normalized intensity curves of the reflected acoustic fields along the *z*-, *x*- and *y*-axes are shown in Fig. 4(c,d). The energy is highly concentrated at the focus point. The peaks of these curves correspond to the focus point (15*a*, 20*a*, 25*a*), in an excellent agreement with the target. In Fig. 4(d), we can obtain that the intensity at the focus point is about 12.89 times larger than that of the incident waves. In addition, subwavelength focusing with the FWHM being 0.73 *λ* is obtained.

As we know, for the normal incidence, 3D-GSL is not necessary to construct a flat lens. This is due to the axial symmetry of the pressure field^28,29^. In this case, the phase profile is written as

8 $$\Phi (x,y)={n}_{0}{k}_{0}(\sqrt{{(x-{x}_{{\rm{f}}})}^{2}+{(y-{y}_{{\rm{f}}})}^{2}+{z}_{{\rm{f}}}^{2}}-{z}_{{\rm{f}}}).$$

Comparing Eqs (7) and (8), we can find that the only difference is the appearance of the term *x* sin *θ*_*i*_ in Eq. (7). This term can be considered as the compensation of the phase from the oblique incidence in *XZ*-plane. Consequently, we can obtain the phase profile of the out-of-plane focus only by superimposing the term *x*sin*θ*_*i*_ to the phase profile of the flat lens with normal incidence. Based on this compensation mechanism, the phase profiles of the metasurface with other wave functions, such as out-of-plane Bessel beams are expected to be obtained simply without using 3D-GSL. However, it must be pointed out that for other complex wave sources, such as a point source, the above analysis may be inconvenient. In this case, 3D-GSL cannot be replaced.

### Broadband modulation of reflected waves

**Figure 5:**
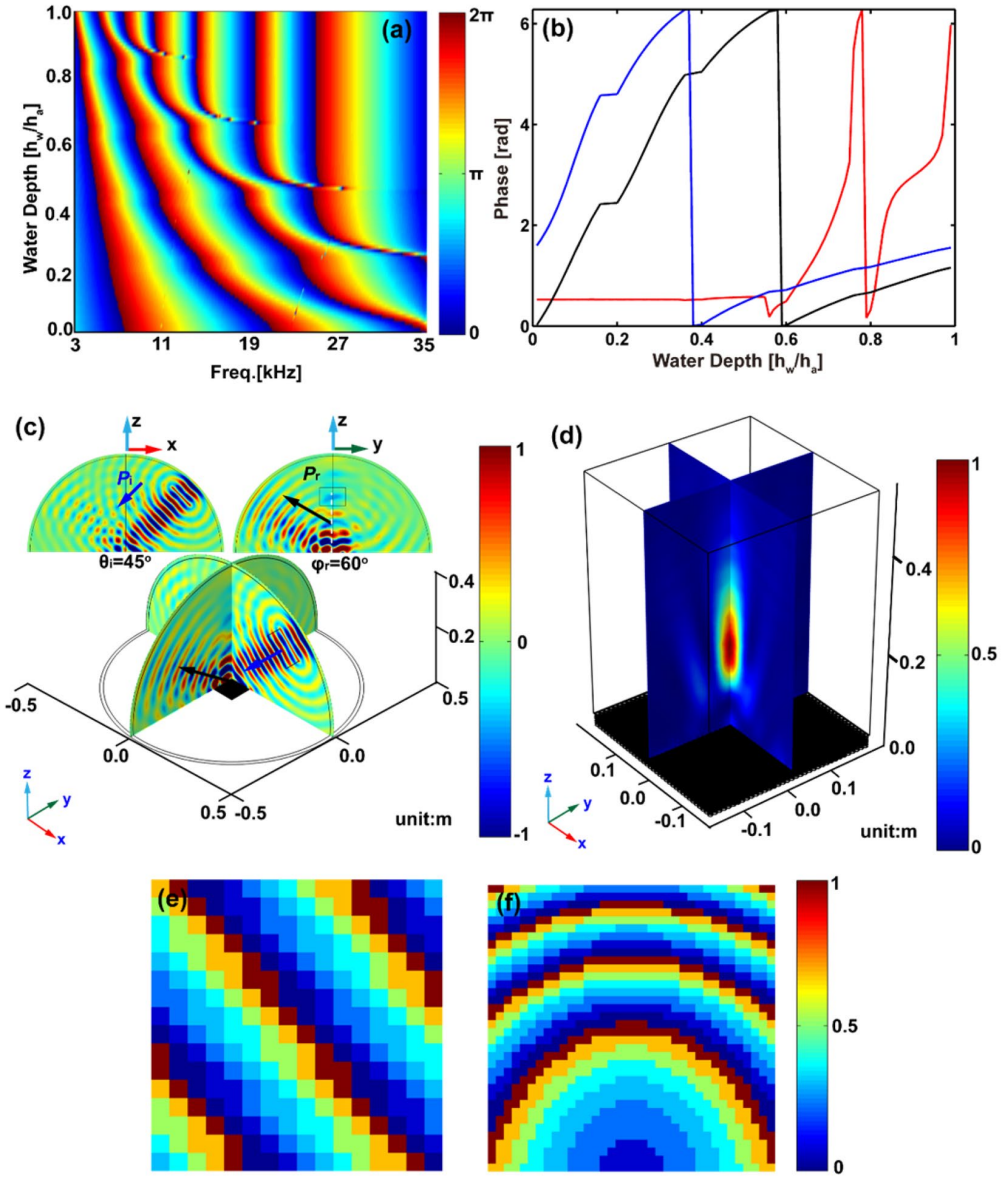
Broadband modulation of reflected waves. (**a**) Shows the phase shift versus frequency and water depth. The color scale represents the phase shift. (**b**) Shows a few horizontal cross-sections of the color plots in panel (a). The red, black and blue lines represent *λ* = *a*(*f* = 34300 Hz), *λ* = 8*a*(*f* = 4287.5 Hz) and *λ* = 10*a*(*f* = 3430 Hz), respectively. Panel (c) shows out-of-plane reflection with the incident angle *θ*_*i*_ = 45° and *φ*_*r*_ = 60° for *λ* = 8*a*. The color scale indicates the normalized reflected waves field. Panel (d) shows a flat lens with the incident angle *θ*_*i*_ = 45° and *z*_*f*_ = 20*a* for *λ* = 8*a*. The color scale indicates the normalized intensity ${|p|}^{2}$. The corresponding distribution of the water depth is shown in (**e**,**f**).

Broadband modulation of wave propagation has been received considerable attention. The proposed metasurface can be operated over a broad band by altering the distribution of the water depth. The variation of the phase shift with the frequency and water depth is plotted in Fig. 5(a). The color scale represents the phase shift. Large phase shifts are found to appear in a wide frequency range from *f* = 34300 Hz (*λ* = *a*) to *f* = 3430 Hz (*λ* = 10*a*). More precisely, Fig. 5(b) illustrates the phase shift as a function of water depth at *λ* = *a*(red line), *λ* = 8*a*(black line) and *λ* = 10*a*(blue line). One can observe that the phase shift covers a full 2*π* span for all frequencies down to *f* = 3430 Hz (*λ* = 10*a*). Therefore, reflected waves can be controlled over a broadband frequency range by changing the distribution of the water depth in the metasurfaces. For example, Fig. 5(c) shows the same modulation of the out-of-plane reflection as Fig. 2(b) but for the operating wavelength *λ* = 8*a*. Another example shown in Fig. 5(d) is focusing the out-of-plane reflected waves at *z*_*f*_ = 20*a* with the incident angle *θ*_*i*_ = 45° and the operating wavelength *λ* = 8*a*. The corresponding distributions of the water depth are shown in Fig. 5(e,f). Numerical simulations agree well with our design. These results confirm that the proposed metasurface works well at other wavelengths.

## Conclusion

In summary, we propose a gradient acoustic metasurface modulating out-of-plane reflection. The fundamental unit cell is a cylindrical hole with gradient annular bulges. The gradient design of the fundamental unit cell can suppress the parasitic reflection efficiently. The phase shift of the reflected waves is changed by filling water into the cylindrical holes, and varies in the whole 2*π* span. By redistributing the water depth, the anomalous out-of-plane reflection with an arbitrary angle can be obtained. Furthermore, subwavelength focusing out of the incident plane at an arbitrary focus point is realized for obliquely incident waves. We also demonstrate that the modulation of the out-of-plane reflected waves is over a wide frequency range.

The proposed concept opens a new direction for engineering wavefront in the three-dimensional space. Due to the flexibility of the proposed metasurface, we can also tailor the reflection for different incident waves, such as cylindrical and spherical waves, just by changing the distribution of the water depth. Moreover, when external devices are used to control the fluid volume, active devices can be expected for tailoring acoustic waves. Finally, we mention that it should be an interesting topic to design a tunable metasurface with nonlocality^27^ to improve the efficiency of the metasurface although it is not a non-trivial task.

## Supplementary information

SI: Modulation of out-of-plane reflected waves by using acoustic metasurfaces with tapered corrugated holes

## Data Availability

All data included in this study are available upon reasonable request by contact with the corresponding author.
