# Supplementary material for: Modulation of out-of-plane reflected waves by using acoustic metasurfaces with tapered corrugated holes
Source: Sci Rep. 2019 Nov 1;9:15856. doi: 10.1038/s41598-019-52441-w (PMC6825153; doi:10.1038/s41598-019-52441-w)
Supplement: Supplementary file 1 — SI: Modulation of out-of-plane reflected waves by using acoustic metasurfaces with tapered corrugated holes [file 41598_2019_52441_MOESM1_ESM.docx]

**Supplementary Material：**

**Modulation of out-of-plane reflected waves by using acoustic metasurfaces with tapered corrugated holes**

Xiao-Shuang Li^1^, Yan-Feng Wang2[[1]](#footnote-1) *, A-Li Chen^1^, Yue-Sheng Wang1,2[[2]](#footnote-2)†

^1^Institute of Engineering Mechanics, Beijing Jiaotong University, Beijing 100044, China

^2^School of Mechanical Engineering, Tianjin University, Tianjin 300072, China

In this supplementary material, we show that the parasitic waves propagating in other directions can be reduced by the gradient design of the metasurfaces intuitively. For comparison, we design a different metasurface with identical annular bulges, as shown in Fig. S1(a). Fig. S1(b) illustrates the variation of the phase profile of the unit with the water depth for $\lambda\text{=}5a$. We can see that the phase shifts cover a $2\pi$span, the same as that shown in Fig. 1(d).

Both metasurfaces are used to reflect waves for and $\theta_{\text{r}}\text{=}0^{\circ}$ with the incident angle $\theta_{\text{i}}\text{=}30^{\circ}$ at $\lambda\text{=}5a$. The related pressure fields are shown in Figs. S1(c) and S1(f). For the gradient metasurface in Fig. S1(f), the parasitic waves almost do not occur in the incident plane, and most of the scattered waves gradually decay in the direction perpendicular to the metasurface. While when the annular bulges are identical, the scattering in the incident plane in Fig. S1(c) is so strong that there exist the parasitic reflected waves $p_{\text{pr}}$ on the incident plane. Moreover, we enlarge the size of the metasurfaces from 16×16 to 24×24 unit cells (~$5\lambda$). The simulation results are presented in Figs. S1(d) and S1(g) for metasurfaces with identical or gradient annular bulges, respectively. It can be observed clearly the parasitic waves propagating to unwanted directions still exist in Fig. S1(d) for the metasurface with identical annular bulges, while the metasurface with gradient annular bulges performs well. This comparison demonstrate that the parasitic reflections are induced by the structure of unit cells itself and independent on the sample size. Next, we consider an incident wave with a fixed beam-width (~$2\lambda$). In this case, the metasurfaces are composed of 24×24 unit cells. The results are depicted in Figs. S1(e) and S1(h). When the incident wave impinges these two metasurfaces, the one with gradient design performs very well and lot of energy is redirected to the target direction. But the parasitic waves still arise on the incident plane for the metasurface without gradient design. These comparisons prompt us to choose the gradient design. Optimal gradient design should be an interesting topic in the future.


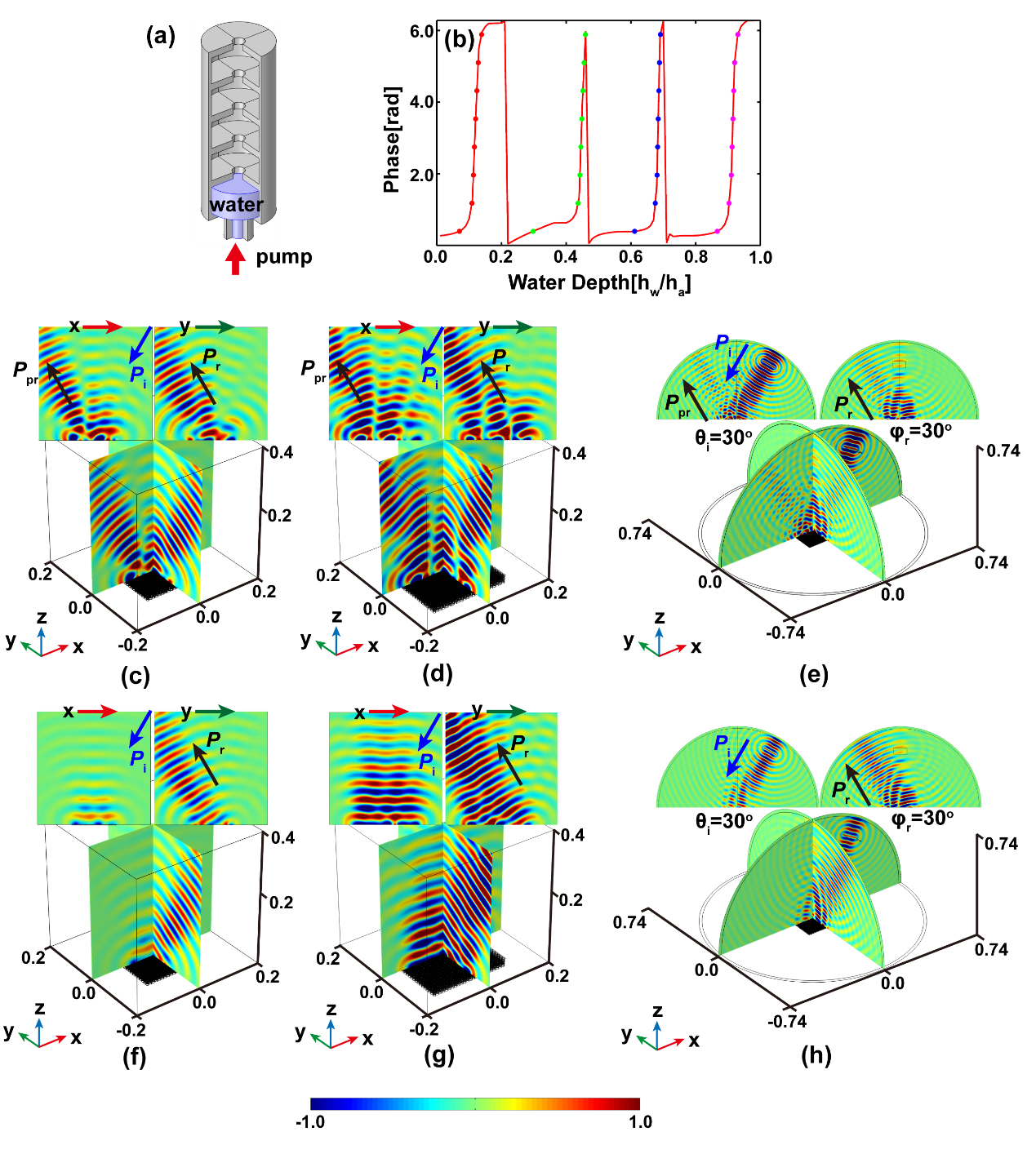


**Figure S1. Comparison of the fundamental units with and without gradient design.** (a) The unit cell without gradient design. (b) The phase profile as a function of the distribution of the water depth. The dots indicate the discrete phase selected for 8 elementary unit cells. Out-of-plane reflections of the metasurfaces without gradient design are depicted in (c) for 16×16 unit cells and (d) for 24×24 unit cells. Panels (f) and (g) show the same results with (c) and (d) but for metasurface with gradient design. When an incident wave with fixed beam width is considered, the out-of-plane reflections are shown in (e) and (h) for the metasurfaces composed of the unit cells without and with gradient design. The blue arrow indicates the incident waves; and the black arrow indicates the reflected waves. The color scale indicates the normalized reflected wave field.

1. * Corresponding author: wangyanfeng@bjtu.edu.cn [↑](#footnote-ref-1)
2. ^†^ Corresponding author: yswang@tju.edu.cn [↑](#footnote-ref-2)
